# Plastic Response of Tracheids in *Pinus pinaster* in a Water-Limited Environment: Adjusting Lumen Size instead of Wall Thickness

**DOI:** 10.1371/journal.pone.0136305

**Published:** 2015-08-25

**Authors:** Ana Carvalho, Cristina Nabais, Joana Vieira, Sergio Rossi, Filipe Campelo

**Affiliations:** 1 CFE–Centre for Functional Ecology, Department of Life Sciences, University of Coimbra, Calçada Martim de Freitas, 3000–456, Coimbra, Portugal; 2 Département des Sciences Fondamentales, Université du Québec à Chicoutimi, Chicoutimi, Canada; 3 Key Laboratory of Vegetation Restoration and Management of Degraded Ecosystems, Provincial Key Laboratory of Applied Botany South China Botanical Garden, Chinese Academy of Sciences, Guangzhou, China; Chinese Academy of Sciences, CHINA

## Abstract

The formation of wood results from cambial activity and its anatomical properties reflect the variability of environmental conditions during the growing season. Recently, it was found that wood density variations in conifers growing under cold-limited environment result from the adjustment of cell wall thickness (CWT) to temperature. Additionally, it is known that intra-annual density fluctuations (IADFs) are formed in response to precipitation after the summer drought. Although IADFs are frequent in Mediterranean conifers no study has yet been conducted to determine if these structures result from the adjustment of lumen diameter (LD) or CWT to soil water availability. Our main objective is to investigate the intra-ring variation of wood anatomical features (LD and CWT) in *Pinus pinaster* Ait. growing under a water-limited environment. We compared the tracheidograms of LD and CWT for the years 2010–2013 in *P*. *pinaster* growing in the west coast of Portugal. Our results suggest a close association between LD and soil moisture content along the growing season, reinforcing the role of water availability in determining tracheid size. Compared with CWT, LD showed a higher intra- and inter-annual variability suggesting its strong adjustment value to variations in water availability. The formation of a latewood IADF appears to be predisposed by higher rates of cell production in spring and triggered by early autumn precipitation. Our findings reinforce the crucial role of water availability on cambial activity and wood formation in Mediterranean conifers, and emphasize the high plasticity of wood anatomical features under Mediterranean climate.

## Introduction

Wood is formed by the vascular cambium that is controlled by intrinsic (e.g. plant hormones) and extrinsic factors (e.g. temperature and precipitation). Thus, the variability of climatic conditions during the growing season can affect cambial activity [1, 2] and tree rings properties by changing the number of xylem cells produced and/or their anatomical properties [3]. The intra-ring variation of wood anatomical parameters, such as wood density [4] and vessel lumen area [5], is correlated with specific climatic conditions and, therefore, can be used as climatic proxies [6–8]. In fact, several studies have found that chronologies of wood anatomical features can have a better climatic signal than tree-ring width chronologies [5, 9].

Xylem anatomy can thus provide valuable information on the environmental conditions controlling cambial activity and cell growth throughout the growing season [10, 11]. For example, *Pinus nigra* Arn. and *Pinus sylvestris* L. from a mesic Mediterranean forest in Spain reduced ring width, tracheid lumen and wall thickness in response to warm and dry summers [12]. Also in Spain, it was observed that tacheid size of *Juniperus thurifera* L. at the beginning of the growing season was strongly correlated with late winter climatic conditions, with a warmer February leading to the formation of larger tracheids [13]. Additionally, it was found that *J*. *thurifera* trees from drier sites presented thicker and smaller tracheids [14]. Adaptations in tracheid anatomy were also observed in a continental site in Switzerland where *P*. *sylvestris* from a dry site showed narrower tree rings and lower latewood proportions compared to a mesic site [15]. These studies indicate that cellular parameters respond to environmental conditions at the time of their formation [16].

Trees are affected by inter- and intra-annual changes in climatic conditions, and the information recorded in xylem is important to understand the effect of climate on wood growth dynamics. Under Mediterranean climate, evergreen trees show two periods of activity, one in spring and another in autumn, associated with mild temperatures and high soil-water availability, and two periods of growth reduction or pause, limited by temperature in winter and water availability in summer [17, 18]. In fact, xylogenesis of conifers under Mediterranean climate showed cambial activity in spring, a reduction or cessation of xylogenesis during summer, followed by cambial resumption in autumn [3, 19, 20]. The autumn resumption of cambial activity is frequently associated with the formation of intra-annual density fluctuations (IADFs), which are visually characterized as earlywood-like cells within latewood [21].

IADFs are often observed in trees growing under Mediterranean climate [22, 23] and their relative position within tree rings can be used to estimate when the triggering factor occurred [21]. Latewood IADFs have been related to above-average precipitation in autumn [24, 25], whereas earlywood IADFs, characterized as latewood-like cells within earlywood, were observed in years with low precipitation in spring [21]. The incorporation of intra-ring anatomical features in ecological and climatological studies can improve and/or reveal new climatic signals [15, 26] and add new information about the meteorological events that took place during the growing season [27]. Wood growth dynamics of *Pinus pinaster* Ait. under Mediterranean climate has been previously investigated [2, 27, 28]. Wider tree-rings were formed due to a higher rate of cell division, rather than by a longer period of xylogenesis [2]. The higher rate of cell division was associated with high water availability in spring, as observed by xylogenesis studies [28] and correlation analysis of tree-ring width and climatic conditions [25, 29]. A study on stem radial variation in *P*. *pinaster* also showed a clear response to water availability in the daily and seasonal pattern, with marked stem contractions and re-hydrations observed in summer and autumn, respectively [30]. The formation of latewood IADFs in *P*. *pinaster* is associated with water availability after the summer drought [25, 28]. This is supported by the positive correlations found between latewood IADFs frequency and September precipitation [24, 31, 32], and by anatomical observations where the resumption of cambial activity and formation of a latewood IADF was observed [28]. All previous studies point out that water availability plays a major role in cambial activity and wood formation under Mediterranean climate. Our main objective was to investigate the intra-ring variation of wood anatomical characteristics, such as lumen diameter (LD) and cell wall thickness (CWT), in *P*. *pinaster* growing under a water-limited environment. To address this, we analyzed the tracheidograms of four consecutive years with different climatic conditions. Tracheidograms represent the variation of a given tracheid feature within a tree ring, and can provide new and additional information on the environmental conditions during the growing season improving our capacity to reconstruct past climate [33, 34]. To this end we compared the intra-ring variation of LD with the intra-seasonal variation of soil water availability.

## Material and Methods

### Study area and tree selection

**Fig 1:**
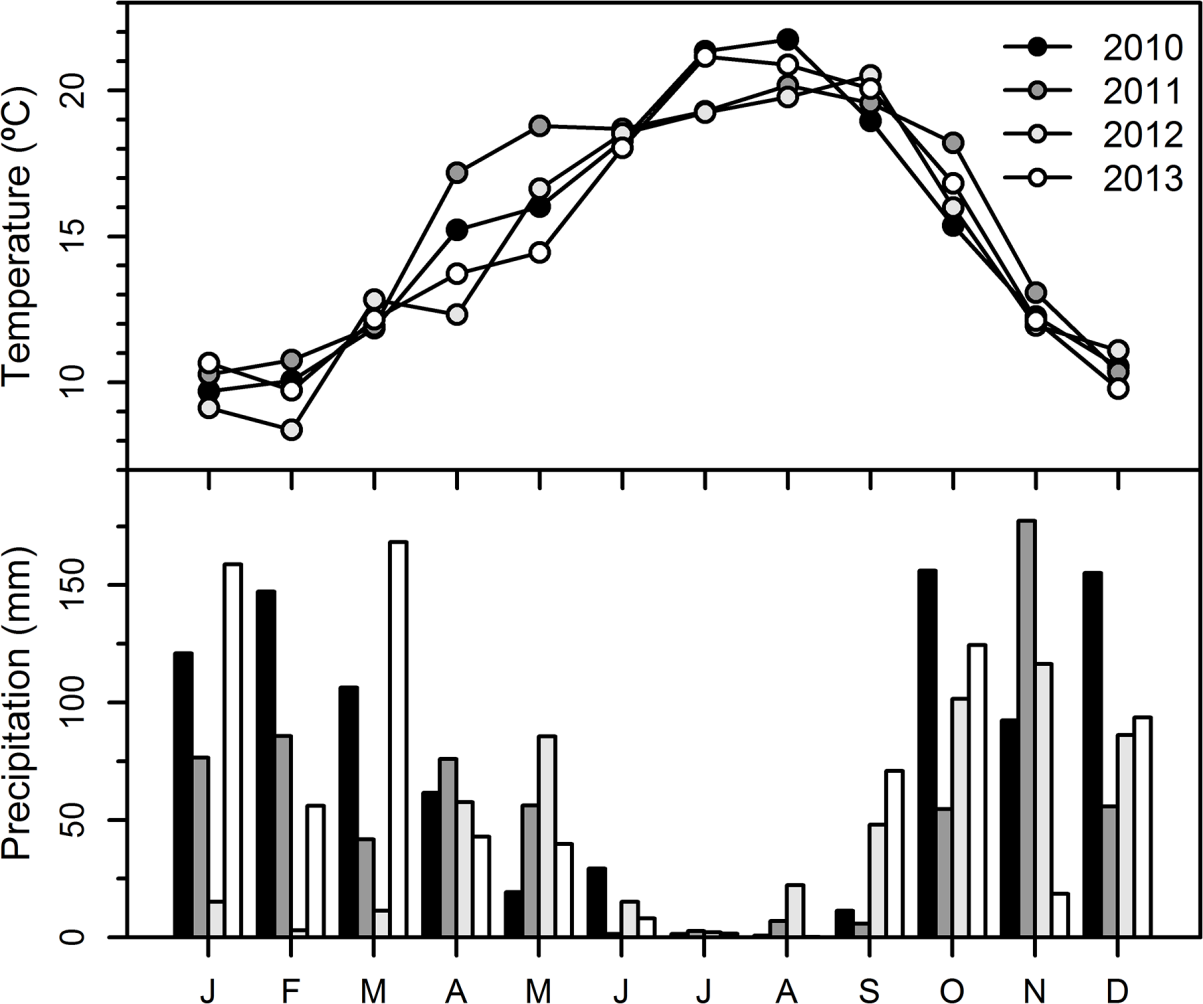
Monthly temperature (°C) and precipitation (mm) for the period 2010–2013 from the closest meteorological station located in Figueira da Foz, at 25 km south from the study area.

The study was carried out in the "Perímetro florestal dunas de Cantanhede" with the permission of the "Instituto da Conservação da Natureza e das Florestas". The study species, *Pinus pinaster*, is not an endangered or protected species. Perímetro florestal dunas de Cantanhede is a plantation of *P*. *pinaster* on sand dunes (40°21’35.15” N, 8°49’10.06” W; 15 m a.s.l.), located in the west coast of Portugal. The study area had a density of approximately 230 trees ha^−1^ and is characterized by dominant and codominant trees with an average age of 45 years. Ten trees without stem or crown anomalies were randomly selected. The climate is typically Mediterranean with oceanic influence, the summer is dry and the precipitation is more abundant in autumn and winter (Fig 1).

Monthly mean temperature and precipitation data were downloaded from the Royal Netherlands Meteorological Institute (http://climexp.knmi.nl), for the closest grid point (CRU TS 3, 0.5° × 0.5°). For the last 30 years, the mean annual temperature was 16° C and the total annual precipitation was 900 mm. For the period 2010–2013, minimum and maximum daily temperatures and total daily precipitation were obtained from the closest meteorological station located in Figueira da Foz, at 25 km south from the study site.

### Sample preparation and measurements

**Fig 2:**
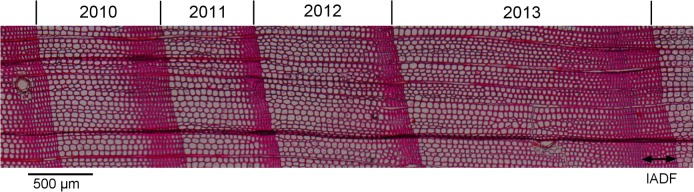
Tree rings of *Pinus pinaster* for the period 2010–2013, with the ring produced in 2013 showing an intra-annual density fluctuation (IADF) in latewood.

One wood microcore per tree (*n* = 10) was collected on the south-facing side of the stem in April 2014 using a Trephor [35]. These microcores were placed in eppendorfs with 50% alcohol solution and stored at 5° C to preserve cells from degradation. In the laboratory, the microcores were dehydrated using a graded alcohol series and D-limonene, and embedded in paraffin [35]. Transverse sections 5–7 μm thick were cut from the samples with a rotary microtome, stained with 1% aqueous safranin and permanently mounted on glass slides with Canada balsam (Eukitt). Digital images were taken at 200 x magnification with a digital camera fixed on a microscope (Fig 2).

Images were analyzed using ImageJ (http://rsbweb.nih.gov/ij/). In each ring, three radial paths were selected to measure radial lumen diameter (LD), cell wall thickness (CWT) and the ratio of LD to CWT (LD/CWT) along the tree rings. For each selected radial file, the number of tracheids (nCells) was determined and tracheids were classified in earlywood and latewood following the Mork's formula [36]. When the LD/CWT ratio was ≥ or < 2, tracheids were classified as earlywood (Ew) or latewood (Lw), respectively. Latewood IADFs are characterized by earlywood-like cells within latewood, thus, we considered that an IADF was formed when the LD/CWT ratio was ≥ 2 after the occurrence of the first latewood tracheid. The “width” of the latewood IADF was given by the number of tracheids inside the latewood showing a LD/CWT ratio ≥ 2 (nIADFs).

### Data Analysis

Tracheidograms represent the intra-ring variation of an anatomical parameter in the radial direction according to its relative position within the ring (S1 Fig; S1 Table) [33]. To allow the comparison of tracheidograms with different number of cells, we normalized the number of cells per tracheidogram to a constant number of cells (S2 Table), using dedicated R functions (based on the tgram package for R [14]) that computes the relative position of each tracheid considering that all rings have the same number of cells. The normalized tracheidogram was 25 cells (16 for earlywood and 9 for latewood). To describe changes in anatomical features (LD, CWT and LD/CWT) we used Generalized Additive Models (GAMs). In this study, the anatomical feature (e.g. LD) was expressed as a function of the position of the cell:

y=α+s(nCell)+ԑ (1)

where *y* is the vector of the anatomical feature (e.g. LD), *nCell* is the vector of the corresponding standardized cell number, *s* is an unspecified smooth function, α is the intercept, and ԑ is the error term. GAMs were also used to investigate intra-ring variability of anatomical features:

ROC(y)=α+s(nCell)+ԑ (2)

where *ROC(y)* is the rate of change of the anatomical feature (in percentage) that represents the increase or decrease of the current tracheid relative to the previous tracheid. GAMs were fitted in R using the mgcv package [37]. Changes in the mean of anatomical variables over the four years (2010–2013) were investigated using repeated-measure ANOVA. When the dependent variable (years) was statistically significant in the ANOVA test (p < 0.01), the Bonferroni corrected pair-wise comparisons were computed to identify specific differences between years. Before running the ANOVA, log transformations of the data were applied when necessary to fulfill the requirements of the statistical analysis for normal distribution and homogeneity of variance.

Monthly soil moisture was estimated using a water balance model [38]. Monthly temperature and precipitation (from the nearest CRU grid point) were used as input data and evapotranspiration, surface runoff, and groundwater flow were estimated by the model. Since soil in the study area is sandy with low organic matter content and low water-holding capacity, the model was run considering that the maximum moisture held by the soil was 0.35 v/v (volume per volume). For each year, LD along the tree ring was visually compared with the monthly soil moisture, considering that the growing season started in March and ended in October, with the transition between earlywood and latewood being set to the middle of June. The timings of xylem formation were based on the observations previously gathered from the same species and in the same site [2, 28]. This approach represents a simplification of the cambial activity by considering that the growing season length is constant and the transition from earlywood to latewood occurs in the same period for different years. Therefore, we assume that the cell division rate of earlywood (latewood) only depends on the number of tracheids differentiated into earlywood (latewood) which can vary from year to year.

## Results

### Climate during 2010–2013

The four studied years presented a lower mean annual temperature than the long-term mean (1984–2013) (Table 1). The average temperature of July and August was higher in 2010 and 2013 than in 2011 and 2012 (Fig 1). Total annual precipitation and the seasonal distribution of precipitation were different between the four years. Contrary to the other years, 2012 had few precipitation events from January to March, while in 2013 precipitation in March was very high (168 mm). In the summer of 2010 and 2013, precipitation was extremely low, while in 2012 it was higher with more than 20 mm in August. Precipitation in September 2012 and 2013 were above the long-term mean. Comparing the four studied years, soil moisture in March and August showed the highest and lowest values in 2013, and in September soil moisture was higher in 2012 (Table 1).

**Table 1:** Mean temperature (T), annual precipitation (P), precipitation in September (Sep P) and soil moisture in March (Mar W), August (Aug W) and September (Sep W) for the long-term mean and for each studied year.

	T (°C)	P (mm)	Sep P (mm)	Mar W (v/v)	Aug W (v/v)	Sep W (v/v)
**1894–2013**	16.0	899	41.3	0.268	0.083	0.114
**2010**	15.1	902	11.3	0.279	0.065	0.057
**2011**	15.7	641	5.8	0.282	0.116	0.103
**2012**	14.7	564	47.9	0.223	0.096	0.150
**2013**	15.0	783	70.9	0.350	0.049	0.114

### Tracheidograms

**Fig 3:**
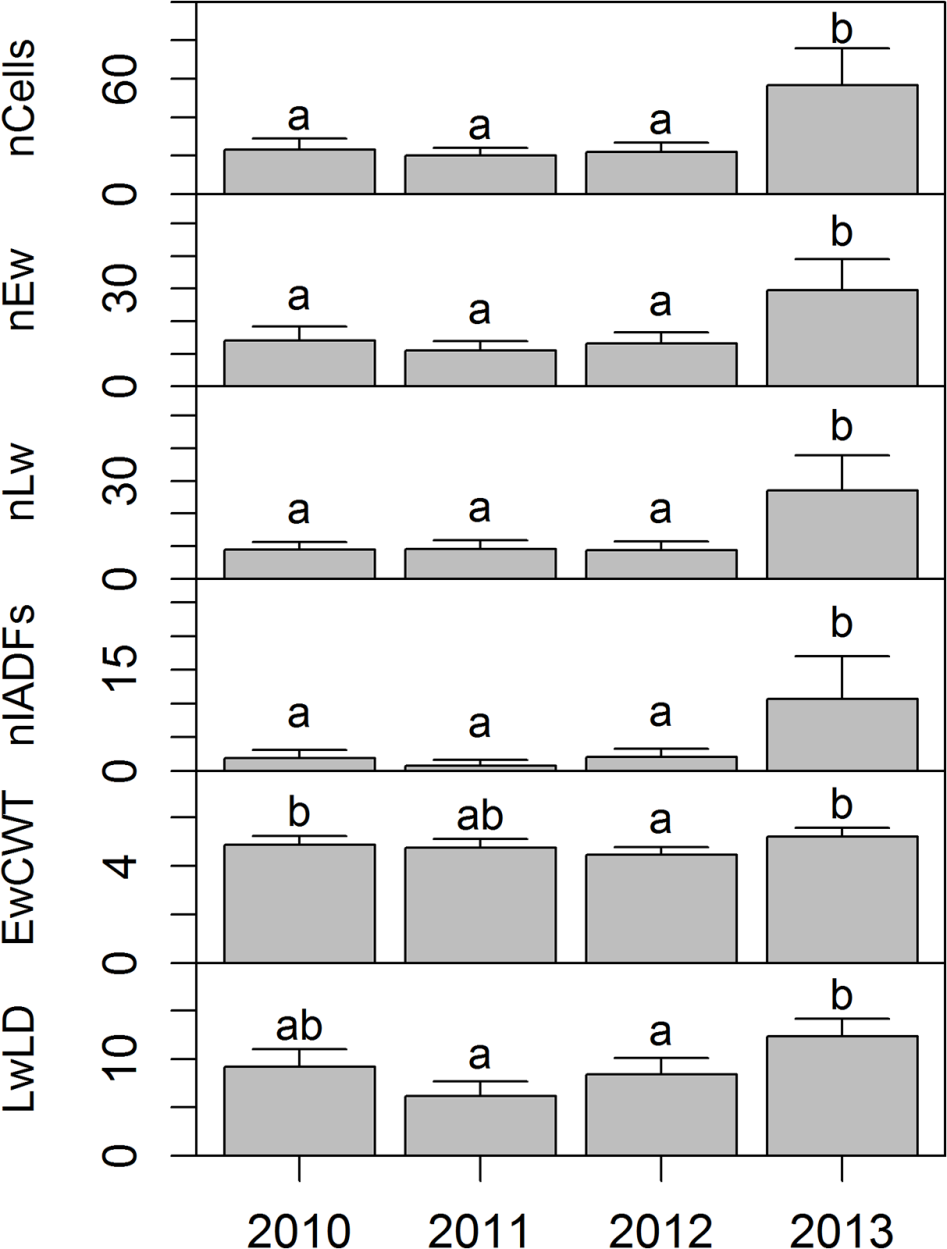
Temporal variation of different anatomical variables for the period 2010–2013. Anatomical variables: number of cells (nCells), number of earlywood tracheids (nEw), number of latewood tracheids (nLw), number of earlywood-like tracheids within the latewood (nIADFs), cell wall thickness of the earlywood (EwCWT) and lumen diameter of latewood (LwLD). Data are the means ± 2SE and different letters indicate significant differences between years.

The total number of tracheids (nCells; *F*_(3,27)_ = 18.57, p < 0.001), earlywood (nEw; *F*_(3,27)_ = 15.29, p < 0.001), latewood tracheids (nLw; *F*_(3,27)_ = 15.76, p < 0.001), and IADFs tracheids (nIADFs; *F*_(3,27)_ = 15.66, p < 0.001) was significantly different between years (Fig 3).

The mean LD of earlywood was not significantly different between years (*F*_(3,27)_ = 4.22, p > 0.01). The mean LD of latewood was significantly different between years (*F*_(3,27)_ = 14.36, p < 0.001), with 2013 showing the highest value (Fig 3). There was a statistically significant difference between years for CWT of earlywood (*F*_(3,27)_ = 9.63, p < 0.001), with the post-hoc test showing that the mean CWT of earlywood in 2013 was higher than in 2012 and 2011. The mean CWT of latewood was not significantly different between years (*F*_(3,27)_ = 3.58, p > 0.01). In 2013, LD and CWT of IADF tracheids presented mean (± SE) values of 18.35 (± 1.25) and 6.17 (± 0.30) μm, respectively, whereas true latewood tracheids had lower values of LD (9.05 ± 0.48 μm) and slightly higher for CWT (7.54 ± 0.17 μm).

**Fig 4:**
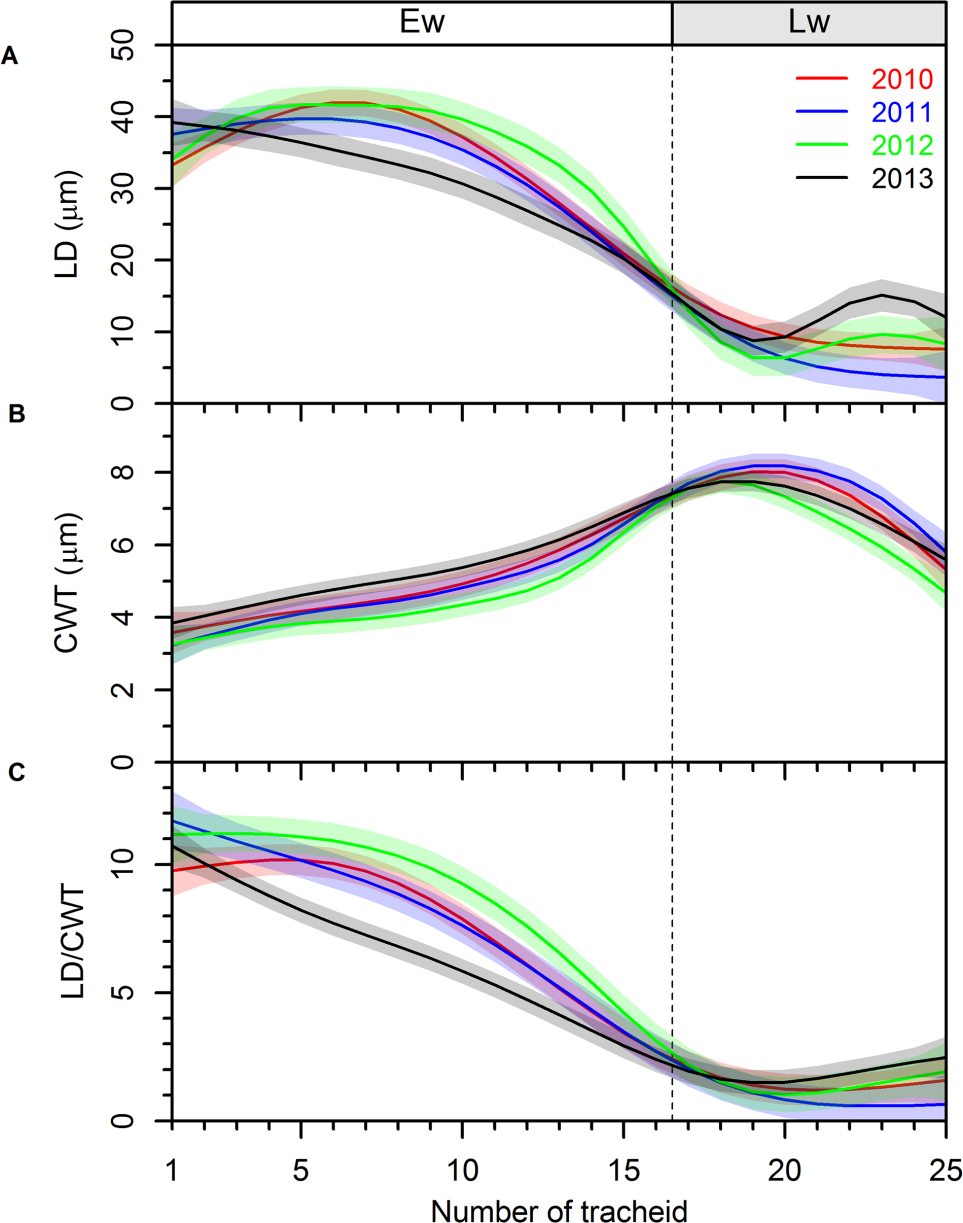
Standardized tracheidograms for the four years (2010–2013). The number of tracheids per ring was set to 25 and each standardize tracheidogram contains 16 tracheids in earlywood (Ew) and 9 in latewood (Lw). A) Lumen diameter (LD); B) Cell wall thickness (CWT); C) Ratio of LD to CWT (LD/CWT). The color lines represent the means and the shaded areas the 95% confidence intervals.

The standardized tracheidograms of anatomical features showed that the intra- and inter-annual variability of LD was higher when compared to CWT (Fig 4A and 4B and S2 Fig). In general, LD gradually decreased from 30–40 to 5–10 μm (Fig 4A), whereas CWT gradually increased from 3–4 to 7–8 μm, decreasing afterwards to 5–6 μm in the last 5 tracheids (Fig 2B). Although LD of the 5–10 tracheids was slightly lower in 2013 than in the other years (Fig 4A), the mean LD of earlywood was not different between years. On the contrary, CWT of earlywood showed differences between years (Fig 3). This is reflected in the lower values of the LD/CWT ratio of the earlywood tracheids of 2013 (Fig 4C). In 2012 and 2013 the decreasing trend of LD was inverted after the beginning of latewood, with the LD of the 22–24 cells in 2013 being higher than in the other years (Fig 2A). Indeed, a clear latewood IADF was produced in 2013, with earlywood-like cells within latewood and a LD/CWT ratio ≥ 2 (Fig 4C and S2 Table). LD can show one or two peaks, one in earlywood and another in latewood, whereas CWT only shows a single peak in the transition between earlywood and latewood.

### Synchronization of LD and soil moisture

**Fig 5:**
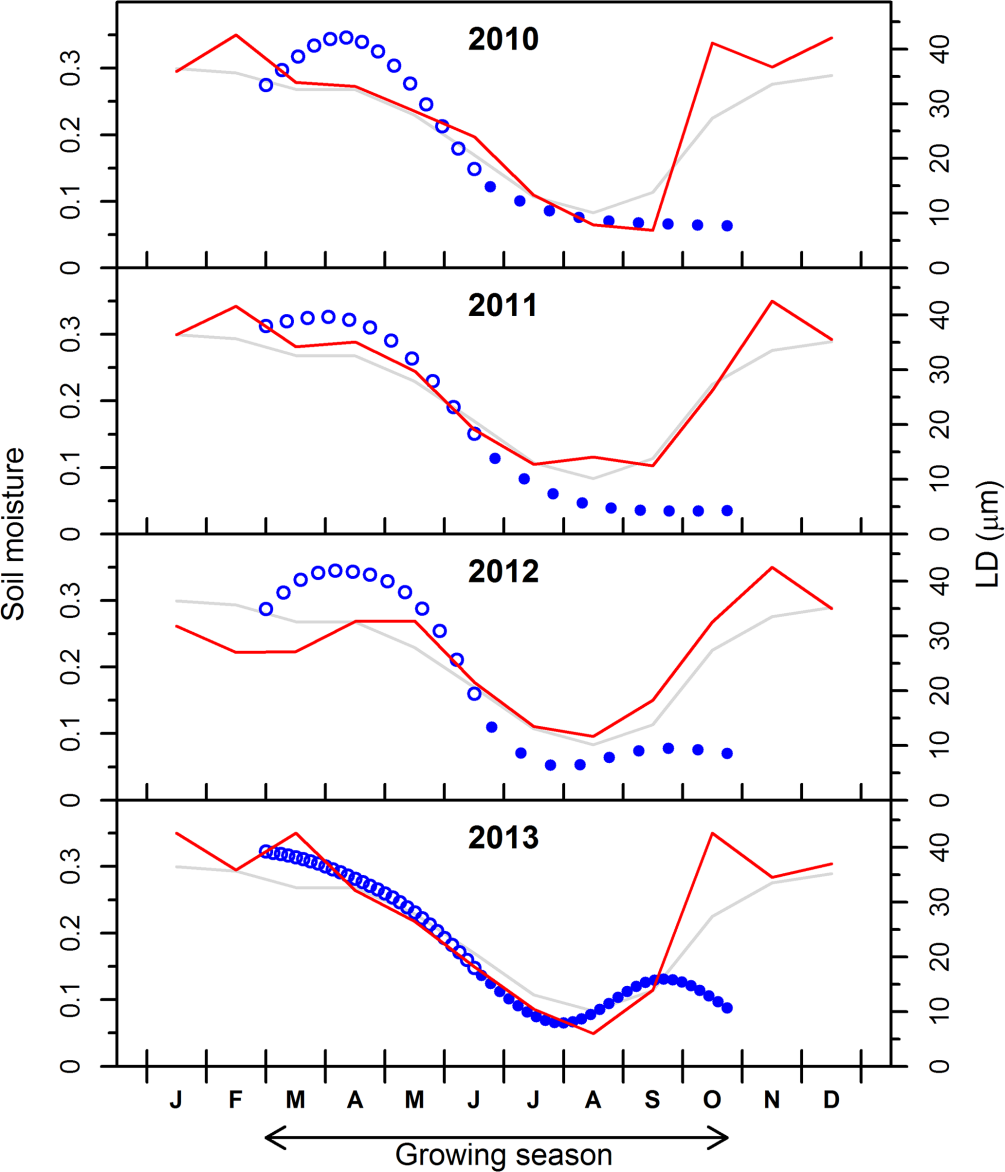
Synchronization of the intra-ring variation of lumen diameter (LD) and sub-seasonal variation of water balance for the period 2010–2013. Open and solid dots indicate the LD of earlywood and latewood tracheids, respectively. Red line indicates the soil moisture for each studied year. Grey line indicates the average soil moisture for the last 30 years.

The seasonal variation in soil moisture is related to the precipitation pattern, with higher moisture in winter and autumn (0.30–0.35 v/v) and lower values in summer (0.05–0.10 v/v) (Fig 5). In summer 2010 and 2013, soil moisture showed the lowest values (< 0.10 v/v) associated with high summer temperatures (Fig 1). In September 2013, soil moisture recovered after the rain events, while in 2010 low soil moisture persisted until the end of September (Fig 5). Analyzing the pattern of soil moisture and LD of tracheids, there is a close association between both variables along the growing season, with the decrease in soil moisture from March to June closely accompanied by a decrease in LD. The increase in soil moisture after the summer drought in 2012 and 2013 was followed by a clear increase in LD only in 2013 (Fig 5).

## Discussion

In this study, we investigated the anatomical features of tracheids in *P*. *pinaster* and its coordination with the climatic conditions over four years in a water-limited environment. The main anatomical differences observed among the four years were a higher number of tracheids in 2013, compared to 2010–2012, and the formation of latewood IADFs in 2012 and 2013, although more evident in 2013. In 2013, LD was 2 times larger for IADF tracheids than for true latewood, whereas the CWT was only 0.2 times thicker for true latewood than for IADF tracheids.

Under Mediterranean climate, cambial activity is mainly controlled by water availability during the growing season [10, 22]. In 2013 the high availability of water at the start of the growing season probably allowed the formation of more tracheids, resulting in a wider tree-ring. Indeed, previous studies found that water availability during spring was related to an increase in the rate of cell production in *P*. *pinaster* [2]. Similarly, the earlywood width in *P*. *halepensis* and *P*. *pinea* was strongly correlated with spring precipitation [21, 39]. Additionally, variations in water availability throughout the growing season can affect the anatomical features of tracheids [13]. Tracheid size represents an important trade-off between hydraulic conductivity and vulnerability to cavitation [40–42], and is highly related to water availability during the cell enlargement phase [13, 14, 24]. In the enlargement phase the pressure potential of the apoplastic water is essential for cell expansion, since it has to be higher than the symplastic water potential for water to enter the expanding cell [43]. This is reinforced by the close association between LD and soil moisture content, with the transition from early- to latewood associated with a decrease in water availability. Our results suggest that the earlywood-latewood transition occurs when soil moisture is <0.15 v/v, which is in agreement with previous studies showing that drier conditions trigger the start of latewood formation [30].

The average earlywood LD was similar among the four studied years. However, when comparing the standardize tracheidograms, between the 5^th^ and 10^th^ tracheid the LD was lower in 2013 compared with the other years. It seems that in 2013, although a higher number of tracheids was produced due to better moisture conditions in the beginning of the growing season, the LD was smaller and CWT larger. This supports the findings of Cuny and co-authors [44], which observed that tracheid diameter is strongly affected by the rate of cell production, with higher rates of cell division associated with narrower tracheids. It is known that auxin plays an important role in the enlargement phase [45]. The concentration of auxin presents a maximum in the cambial zone decreasing towards the differentiating xylem where it reaches a low and stable value close to the transition between enlargement and cell-wall thickening cells [46]. The width of this gradient determines the width of the enlargement zone and determines the time cells spend in that phase [47]. Under higher rates of cell division, the newly divided cells push the previous cells out of the enlargement zone, towards the cell wall thickening zone, decreasing the time spent by cells in enlargement, and consequently reducing LD. This suggests that LD is not only determined by soil water availability but also by the amount of time cells spend in the enlargement zone, which in turn depends on the rate of cell production.

Our results showed a higher inter-annual variability of LD, compared to CWT, suggesting that wood anatomical variations among different years were mainly caused by changes in LD, with an apparent coordination with soil-water availability along the growing season. Contrasting results were found by Fonti and co-authors [34], who reported that in trees of *Larix sibirica* Ldb. growing in southern Siberia, the adjustment of xylem was mainly reflected in changes of CWT and highly dependent on temperature. The differences observed in the response of trees growing under water- and temperature-limited environments reflect the dominant environmental constraints to cambial activity. In cold-limited environments trees have to guarantee that lignification ends before the start of winter [48, 49]. Therefore, the adjustment of tracheid features in cold environments occurs in the lignification phase and not in the enlargement phase, as observed in water-limited environments. Nonetheless, Cuny and co-authors [44] showed that changes in CWT are driven by cell size, with the amount of material deposited per cell being almost constant along most of the tree ring. Thus, the increase in CWT in latewood cells is the result of a similar amount of lignin placed in a smaller lumen volume. This relation is noticeably seen in our results when comparing the opposite patterns of the tracheidograms of LD and CWT.

The formation of a latewood IADF was clearly observed in 2013 and marginally in 2012. Previous studies showed a strong correlation between latewood IADFs and September precipitation [24, 25, 29, 31, 50]. The release of water stress after the summer drought can induce the resumption of cambial activity and consequent entrance of the new cells into the enlargement zone. The recovery of the tree water status [2] permits cells to expand more than true latewood cells. As a result, in 2013 a clear latewood IADF was formed, with 11 out of 27 latewood cells to be considered as earlywood-like cells (LD/CWT ≥ 2). This finding suggests that the combination of a dry summer and wet early autumn is necessary to trigger the formation of a latewood IADF [2, 24]. This is reinforced by the fact that in 2010 and 2011, with low precipitation events in September, no latewood IADF was formed. In 2012, although there were rainfall events in September, only a marginal latewood IADF was formed, suggesting that other factors are also involved in IADF formation [31]. Previous studies showed that wider tree rings are more prone to form IADFs than smaller rings [31]. We believe that the formation of a latewood IADF in 2013 was predisposed by the higher rates of cell production and triggered by the rainfall events after the summer drought. The higher soil water availability during the start of the growing season induces higher rates of cell production which, in turn, increases the number of cells under enlargement after the summer drought, predisposing the formation of an IADF in response to September precipitation.

*Pinus pinaster* trees show a high capacity to adjust the size of their tracheids to the current soil water content. Conifers growing under water-limited environments appear to adjust LD, while in temperature-limited environments conifers adjust CWT [34] We hypothesized that the formation of latewood IADFs is predisposed by high rates of cell production and triggered by the combination of a dry summer followed by a wet early autumn. However, our observations are only based in 4 years, limiting our conclusions. Further experimental studies are thus necessary to understand (i) if soil moisture content along the growing season is the major driving force of LD; (ii) if higher moisture content in the beginning of the growing season actually leads to a higher rate of cell production; and (iii) if the formation of latewood IADFs is predisposed by higher rates of cell production and triggered by precipitation events after the summer drought.

## Supporting Information

S1 Fig Tracheidograms for the period 2010–2013, the number of tracheids corresponds to the mean number of cells. A) Lumen diameter (LD); B) Cell wall thickness (CWT); C) Ratio of LD to CWT (LD/CWT). The color lines represent the means, the shaded areas the 95% confidence intervals and filled dots represent latewood tracheids.
(TIF)

S2 Fig Rate of variation (in percentage) of standardized tracheidograms, for the period 2010–2013. The number of tracheids per ring was set to 25 and each standardize tracheidogram contains 16 tracheids in earlywood (Ew) and 9 in latewood (Lw). A) Lumen diameter (LD); B) Variation of cell wall thickness (CWT); C) Ratio of LD to CWT (LD/CWT). The color lines represent the means, the shaded areas the 95% confidence intervals.
(TIF)

S1 Table Data used in S1 Fig. Mean ± SE of lumen diameter (LD), cell wall thickness (CWT) and ratio of LD to CWT (LD/CWT) in function of cell position for the period 2010–2013.
(PDF)

S2 Table Data used in Fig 4. Mean ± SE of lumen diameter (LD), cell wall thickness (CWT) and ratio of LD to CWT (LD/CWT) in function of the standardized cell position for the period 2010–2013.
(PDF)
