# Supplementary figures and images for: Plastic Response of Tracheids in Pinus pinaster in a Water-Limited Environment: Adjusting Lumen Size instead of Wall Thickness
Source: PLoS One. 2015 Aug 25;10(8):e0136305. doi: 10.1371/journal.pone.0136305 (PMC4549277; doi:10.1371/journal.pone.0136305)

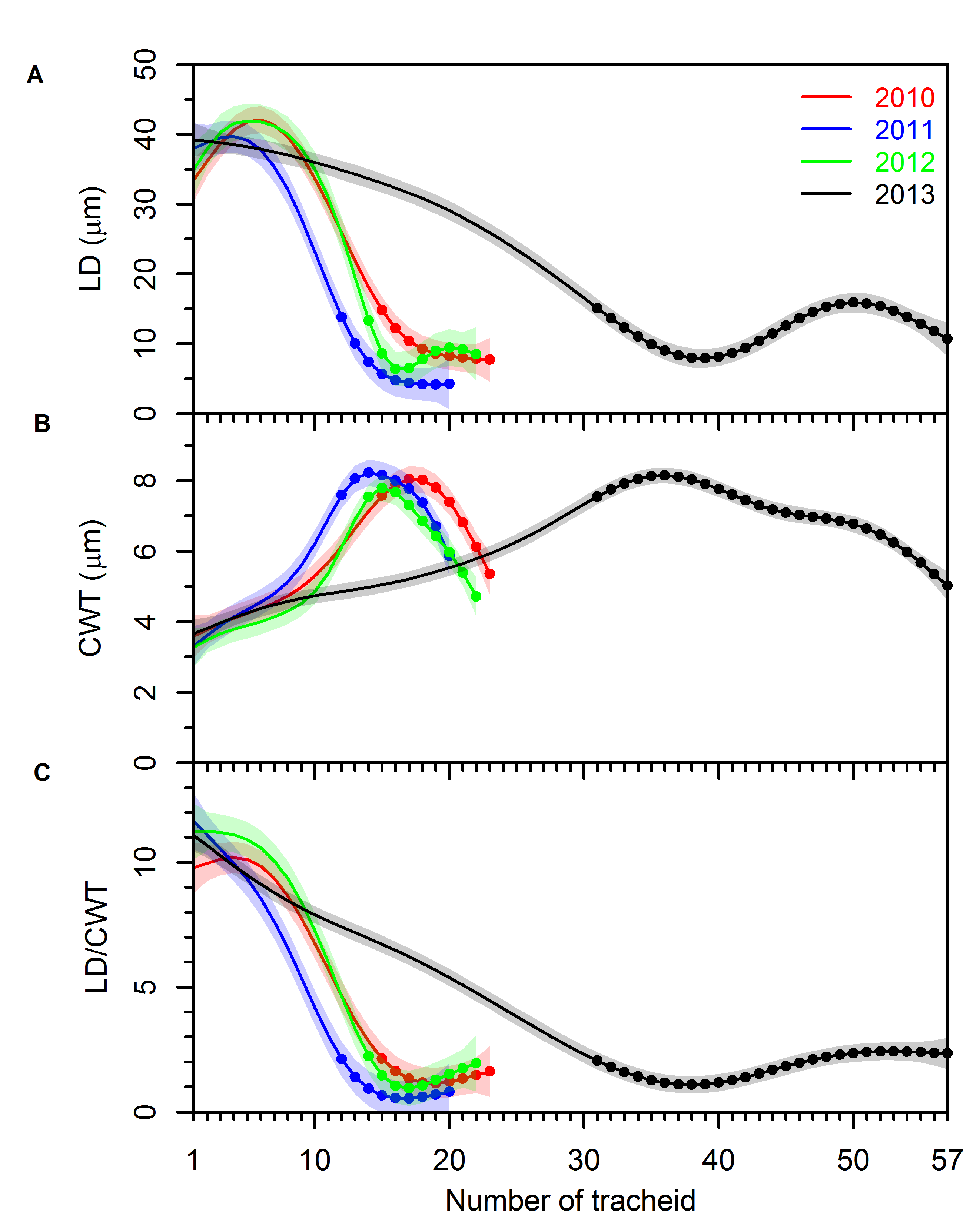

Supplement: S1 Fig — A) Lumen diameter (LD); B) Cell wall thickness (CWT); C) Ratio of LD to CWT (LD/CWT). The color lines represent the means, the shaded areas the 95% confidence intervals and filled dots represent latewood tracheids. (TIF) [file pone.0136305.s001.tif]

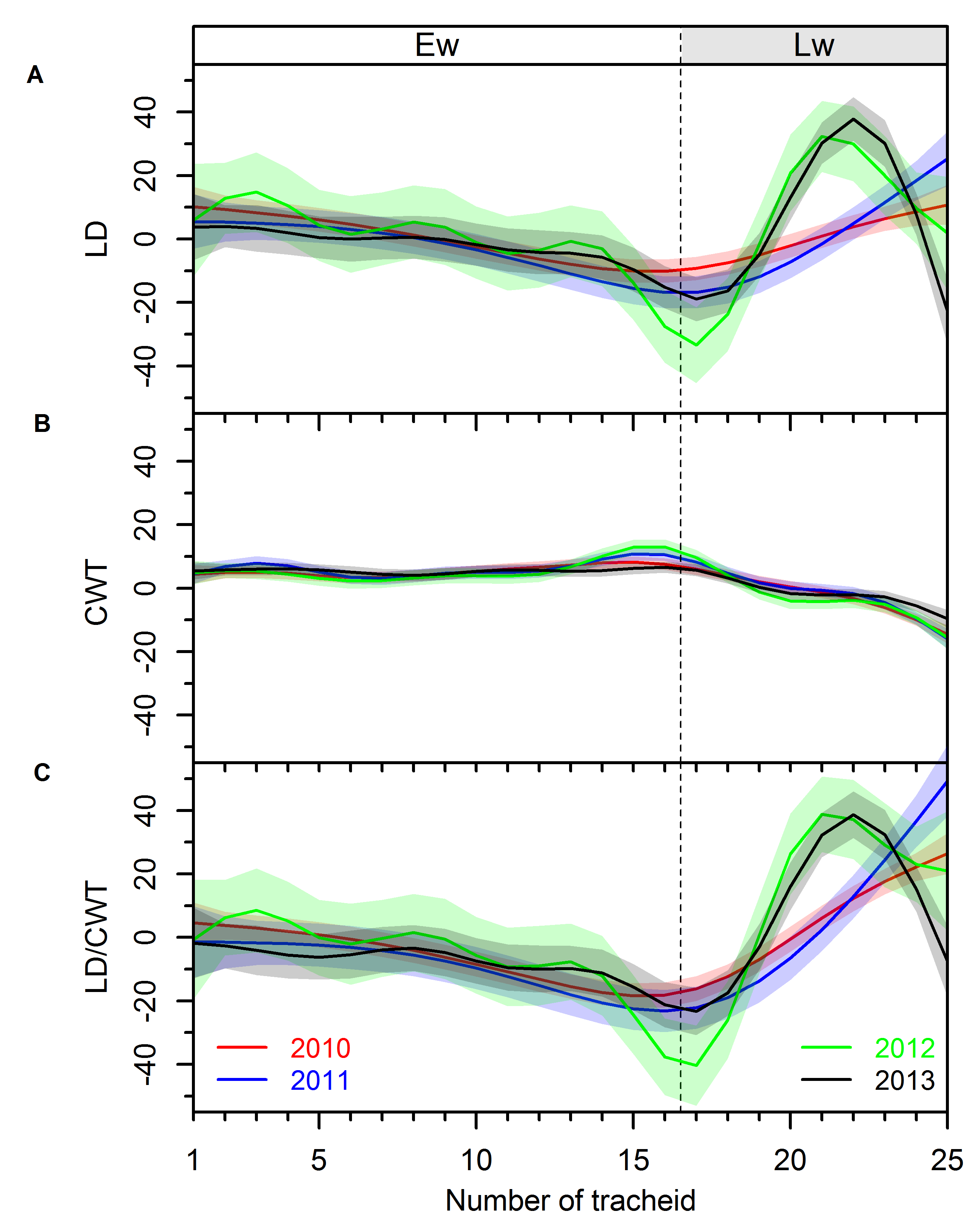

Supplement: S2 Fig — The number of tracheids per ring was set to 25 and each standardize tracheidogram contains 16 tracheids in earlywood (Ew) and 9 in latewood (Lw). A) Lumen diameter (LD); B) Variation of cell wall thickness (CWT); C) Ratio of LD to CWT (LD/CWT). The color lines represent the means, the shaded areas the 95% confidence intervals. (TIF) [file pone.0136305.s002.tif]
