# Supplementary material for: Plastic Response of Tracheids in Pinus pinaster in a Water-Limited Environment: Adjusting Lumen Size instead of Wall Thickness
Source: PLoS One. 2015 Aug 25;10(8):e0136305. doi: 10.1371/journal.pone.0136305 (PMC4549277; doi:10.1371/journal.pone.0136305)
Supplement: S1 Table — Mean ± SE of lumen diameter (LD), cell wall thickness (CWT) and ratio of LD to CWT (LD/CWT) in function of cell position for the period 2010–2013. (PDF) [file pone.0136305.s003.pdf]

Standardized cell number	LD (μm)				CWT (μm)				LD/CWT			
	2010	2011	2012	2013	2010	2011	2012	2013	2010	2011	2012	2013
	Mean ± SE	Mean ± SE	Mean ± SE	Mean ± SE	Mean ± SE	Mean ± SE	Mean ± SE	Mean ± SE	Mean ± SE	Mean ± SE	Mean ± SE	Mean ± SE
1	33.46 ± 3.13	37.99 ± 3.62	34.91 ± 3.84	39.27 ± 2.39	3.59 ± 0.59	3.30 ± 0.57	3.27 ± 0.56	3.67 ± 0.40	9.79 ± 1.02	11.66 ± 1.14	11.25 ± 1.13	11.07 ± 0.62
2	36.18 ± 2.15	38.86 ± 2.48	37.94 ± 2.52	39.02 ± 1.87	3.77 ± 0.41	3.61 ± 0.38	3.49 ± 0.36	3.83 ± 0.31	9.97 ± 0.72	11.09 ± 0.80	11.24 ± 0.77	10.65 ± 0.50
3	38.67 ± 1.98	39.51 ± 2.31	40.27 ± 2.64	38.76 ± 1.50	3.94 ± 0.38	3.89 ± 0.39	3.67 ± 0.38	3.98 ± 0.25	10.13 ± 0.64	10.53 ± 0.72	11.21 ± 0.72	10.24 ± 0.41
4	40.64 ± 1.95	39.69 ± 2.28	41.53 ± 2.50	38.47 ± 1.34	4.10 ± 0.37	4.14 ± 0.38	3.80 ± 0.36	4.13 ± 0.22	10.20 ± 0.63	9.95 ± 0.71	11.11 ± 0.71	9.83 ± 0.36
5	41.84 ± 1.94	39.15 ± 2.29	41.91 ± 2.52	38.16 ± 1.34	4.24 ± 0.37	4.35 ± 0.38	3.90 ± 0.37	4.27 ± 0.23	10.12 ± 0.63	9.31 ± 0.71	10.91 ± 0.71	9.44 ± 0.35
6	42.09 ± 1.94	37.74 ± 2.25	41.76 ± 2.51	37.79 ± 1.40	4.38 ± 0.37	4.56 ± 0.37	4.00 ± 0.36	4.39 ± 0.24	9.85 ± 0.63	8.54 ± 0.70	10.57 ± 0.70	9.07 ± 0.35
7	41.32 ± 1.91	35.37 ± 2.26	41.18 ± 2.48	37.37 ± 1.41	4.54 ± 0.36	4.80 ± 0.38	4.14 ± 0.36	4.50 ± 0.24	9.35 ± 0.62	7.62 ± 0.70	10.06 ± 0.69	8.72 ± 0.36
8	39.57 ± 1.92	32.06 ± 2.24	39.99 ± 2.53	36.91 ± 1.38	4.73 ± 0.37	5.13 ± 0.37	4.30 ± 0.37	4.60 ± 0.23	8.65 ± 0.62	6.55 ± 0.69	9.35 ± 0.70	8.40 ± 0.35
9	36.97 ± 1.91	27.92 ± 2.25	37.98 ± 2.47	36.39 ± 1.32	4.98 ± 0.36	5.59 ± 0.38	4.52 ± 0.36	4.68 ± 0.22	7.77 ± 0.61	5.38 ± 0.70	8.42 ± 0.69	8.10 ± 0.34
10	33.69 ± 1.91	23.21 ± 2.24	35.01 ± 2.52	35.84 ± 1.30	5.29 ± 0.36	6.21 ± 0.38	4.86 ± 0.37	4.75 ± 0.22	6.77 ± 0.61	4.19 ± 0.69	7.29 ± 0.70	7.83 ± 0.34
11	29.92 ± 1.92	18.34 ± 2.24	30.94 ± 2.49	35.25 ± 1.31	5.68 ± 0.37	6.93 ± 0.38	5.39 ± 0.36	4.82 ± 0.22	5.71 ± 0.62	3.07 ± 0.69	5.99 ± 0.69	7.57 ± 0.34
12	25.89 ± 1.90	13.80 ± 2.25	25.67 ± 2.49	34.64 ± 1.34	6.14 ± 0.36	7.60 ± 0.38	6.12 ± 0.36	4.88 ± 0.23	4.65 ± 0.61	2.12 ± 0.70	4.63 ± 0.69	7.32 ± 0.34
13	21.85 ± 1.92	10.06 ± 2.24	19.46 ± 2.52	34.00 ± 1.36	6.65 ± 0.37	8.05 ± 0.37	6.91 ± 0.37	4.94 ± 0.23	3.67 ± 0.62	1.41 ± 0.69	3.33 ± 0.70	7.08 ± 0.34
14	18.07 ± 1.91	7.37 ± 2.26	13.31 ± 2.47	33.35 ± 1.35	7.14 ± 0.36	8.22 ± 0.38	7.54 ± 0.36	5.01 ± 0.23	2.81 ± 0.61	0.93 ± 0.70	2.23 ± 0.69	6.84 ± 0.34
15	14.81 ± 1.91	5.68 ± 2.25	8.62 ± 2.53	32.66 ± 1.32	7.57 ± 0.36	8.17 ± 0.37	7.80 ± 0.37	5.09 ± 0.22	2.13 ± 0.61	0.67 ± 0.70	1.46 ± 0.70	6.59 ± 0.34
16	12.24 ± 1.92	4.77 ± 2.29	6.38 ± 2.48	31.93 ± 1.30	7.89 ± 0.37	8.01 ± 0.38	7.67 ± 0.36	5.17 ± 0.22	1.64 ± 0.62	0.55 ± 0.71	1.05 ± 0.69	6.34 ± 0.34
17	10.41 ± 1.91	4.35 ± 2.28	6.47 ± 2.51	31.16 ± 1.31	8.04 ± 0.36	7.78 ± 0.38	7.30 ± 0.36	5.27 ± 0.22	1.33 ± 0.62	0.54 ± 0.71	0.95 ± 0.70	6.07 ± 0.34
18	9.25 ± 1.94	4.19 ± 2.31	7.78 ± 2.52	30.32 ± 1.33	8.02 ± 0.37	7.37 ± 0.39	6.86 ± 0.37	5.37 ± 0.23	1.18 ± 0.63	0.60 ± 0.72	1.07 ± 0.71	5.78 ± 0.34
19	8.58 ± 1.94	4.18 ± 2.48	9.01 ± 2.50	29.40 ± 1.35	7.81 ± 0.37	6.71 ± 0.38	6.43 ± 0.36	5.48 ± 0.23	1.16 ± 0.63	0.70 ± 0.80	1.29 ± 0.71	5.48 ± 0.34
20	8.22 ± 1.95	4.24 ± 3.62	9.46 ± 2.64	28.41 ± 1.35	7.40 ± 0.37	5.86 ± 0.57	5.96 ± 0.38	5.61 ± 0.23	1.22 ± 0.63	0.81 ± 1.14	1.52 ± 0.72	5.18 ± 0.34
21	8.01 ± 1.98		9.18 ± 2.52	27.34 ± 1.33	6.82 ± 0.38		5.39 ± 0.36	5.75 ± 0.23	1.33 ± 0.64		1.74 ± 0.77	4.86 ± 0.34
22	7.86 ± 2.15		8.53 ± 3.84	26.19 ± 1.31	6.12 ± 0.41		4.71 ± 0.56	5.90 ± 0.22	1.47 ± 0.72		1.95 ± 1.13	4.53 ± 0.34
23	7.72 ± 3.13			24.96 ± 1.31	5.36 ± 0.59			6.06 ± 0.22	1.63 ± 1.02			4.20 ± 0.34
24				23.67 ± 1.32				6.24 ± 0.22				3.88 ± 0.34
25				22.32 ± 1.34				6.44 ± 0.23				3.55 ± 0.34
26				20.93 ± 1.35				6.65 ± 0.23				3.23 ± 0.34
27				19.50 ± 1.34				6.87 ± 0.23				2.92 ± 0.34
28				18.05 ± 1.32				7.09 ± 0.22				2.62 ± 0.34
29				16.60 ± 1.31				7.32 ± 0.22				2.34 ± 0.34
30				15.16 ± 1.32				7.54 ± 0.22				2.07 ± 0.34
31				13.76 ± 1.34				7.74 ± 0.23				1.83 ± 0.34
32				12.43 ± 1.35				7.91 ± 0.23				1.61 ± 0.34
33				11.19 ± 1.34				8.04 ± 0.23				1.43 ± 0.34
34				10.09 ± 1.32				8.12 ± 0.22				1.28 ± 0.34
35				9.18 ± 1.31				8.15 ± 0.22				1.17 ± 0.34
36				8.49 ± 1.31				8.13 ± 0.22				1.10 ± 0.34
37				8.05 ± 1.33				8.06 ± 0.23				1.07 ± 0.34
38				7.88 ± 1.35				7.95 ± 0.23				1.08 ± 0.34
39				8.01 ± 1.35				7.81 ± 0.23				1.14 ± 0.34
40				8.41 ± 1.33				7.66 ± 0.23				1.22 ± 0.34
41				9.06 ± 1.31				7.50 ± 0.22				1.33 ± 0.34
42				9.92 ± 1.30				7.36 ± 0.22				1.47 ± 0.34
43				10.92 ± 1.32				7.23 ± 0.22				1.61 ± 0.34
44				12.00 ± 1.35				7.13 ± 0.23				1.76 ± 0.34
45				13.06 ± 1.36				7.06 ± 0.23				1.90 ± 0.34
46				14.04 ± 1.34				7.00 ± 0.23				2.04 ± 0.34
47				14.86 ± 1.31				6.95 ± 0.22				2.15 ± 0.34
48				15.48 ± 1.30				6.91 ± 0.22				2.25 ± 0.34
49				15.84 ± 1.32				6.84 ± 0.22				2.33 ± 0.34
50				15.92 ± 1.38				6.75 ± 0.23				2.38 ± 0.35
51				15.73 ± 1.41				6.61 ± 0.24				2.41 ± 0.36
52				15.28 ± 1.40				6.43 ± 0.24				2.43 ± 0.35
53				14.61 ± 1.34				6.21 ± 0.23				2.43 ± 0.35
54				13.76 ± 1.34				5.94 ± 0.22				2.41 ± 0.36
55				12.79 ± 1.50				5.65 ± 0.25				2.39 ± 0.41
56				11.74 ± 1.87				5.33 ± 0.31				2.37 ± 0.50
57				10.67 ± 2.39				5.01 ± 0.40				2.34 ± 0.62
