# Supplementary material for: Plastic Response of Tracheids in Pinus pinaster in a Water-Limited Environment: Adjusting Lumen Size instead of Wall Thickness
Source: PLoS One. 2015 Aug 25;10(8):e0136305. doi: 10.1371/journal.pone.0136305 (PMC4549277; doi:10.1371/journal.pone.0136305)
Supplement: S2 Table — Mean ± SE of lumen diameter (LD), cell wall thickness (CWT) and ratio of LD to CWT (LD/CWT) in function of the standardized cell position for the period 2010–2013. (PDF) [file pone.0136305.s004.pdf]

Standardized cell number	LD (μm)				CWT (μm)				LD/CWT			
	2010	2011	2012	2013	2010	2011	2012	2013	2010	2011	2012	2013
	Mean ± SE	Mean ± SE	Mean ± SE	Mean ± SE	Mean ± SE	Mean ± SE	Mean ± SE	Mean ± SE	Mean ± SE	Mean ± SE	Mean ± SE	Mean ± SE
1	33.28 ± 3.02	37.56 ± 3.67	34.06 ± 3.89	39.20 ± 3.28	3.58 ± 0.58	3.23 ± 0.53	3.25 ± 0.52	3.84 ± 0.44	9.76 ± 0.99	11.70 ± 1.15	11.17 ± 1.11	10.72 ± 0.81
2	35.71 ± 2.12	38.32 ± 2.62	37.29 ± 2.51	38.70 ± 2.09	3.75 ± 0.40	3.47 ± 0.35	3.44 ± 0.34	4.04 ± 0.30	9.93 ± 0.71	11.30 ± 0.85	11.20 ± 0.78	10.04 ± 0.57
3	37.99 ± 1.89	39.00 ± 2.29	39.81 ± 2.63	38.08 ± 2.25	3.91 ± 0.37	3.71 ± 0.35	3.60 ± 0.35	4.24 ± 0.28	10.09 ± 0.62	10.90 ± 0.72	11.21 ± 0.70	9.39 ± 0.51
4	39.91 ± 1.86	39.50 ± 2.23	41.21 ± 2.55	37.31 ± 2.16	4.05 ± 0.37	3.93 ± 0.35	3.74 ± 0.34	4.44 ± 0.28	10.18 ± 0.60	10.53 ± 0.69	11.18 ± 0.69	8.77 ± 0.50
5	41.29 ± 1.84	39.75 ± 2.21	41.67 ± 2.44	36.39 ± 2.06	4.17 ± 0.36	4.11 ± 0.33	3.83 ± 0.33	4.61 ± 0.27	10.18 ± 0.60	10.16 ± 0.69	11.09 ± 0.68	8.22 ± 0.49
6	41.99 ± 1.85	39.68 ± 2.22	41.68 ± 2.55	35.40 ± 2.18	4.29 ± 0.36	4.24 ± 0.34	3.89 ± 0.34	4.77 ± 0.27	10.04 ± 0.60	9.77 ± 0.69	10.93 ± 0.68	7.72 ± 0.49
7	41.91 ± 1.82	39.25 ± 2.19	41.61 ± 2.47	34.39 ± 2.10	4.41 ± 0.36	4.34 ± 0.33	3.96 ± 0.33	4.91 ± 0.27	9.74 ± 0.59	9.35 ± 0.68	10.68 ± 0.67	7.26 ± 0.49
8	41.05 ± 1.81	38.42 ± 2.18	41.40 ± 2.47	33.35 ± 2.10	4.55 ± 0.36	4.46 ± 0.33	4.06 ± 0.33	5.05 ± 0.27	9.27 ± 0.59	8.85 ± 0.68	10.34 ± 0.67	6.81 ± 0.49
9	39.45 ± 1.82	37.15 ± 2.19	40.80 ± 2.53	32.16 ± 2.17	4.72 ± 0.36	4.62 ± 0.34	4.19 ± 0.34	5.20 ± 0.27	8.64 ± 0.59	8.28 ± 0.68	9.87 ± 0.67	6.34 ± 0.49
10	37.22 ± 1.81	35.40 ± 2.17	39.62 ± 2.46	30.69 ± 2.08	4.92 ± 0.35	4.82 ± 0.33	4.35 ± 0.33	5.38 ± 0.27	7.87 ± 0.59	7.62 ± 0.67	9.26 ± 0.67	5.84 ± 0.48
11	34.47 ± 1.82	33.18 ± 2.18	37.95 ± 2.50	28.90 ± 2.13	5.18 ± 0.36	5.03 ± 0.34	4.52 ± 0.33	5.59 ± 0.27	7.01 ± 0.59	6.88 ± 0.67	8.50 ± 0.67	5.30 ± 0.49
12	31.34 ± 1.82	30.50 ± 2.18	35.88 ± 2.52	26.91 ± 2.15	5.49 ± 0.36	5.27 ± 0.34	4.74 ± 0.33	5.85 ± 0.27	6.09 ± 0.59	6.07 ± 0.67	7.59 ± 0.67	4.73 ± 0.49
13	27.94 ± 1.81	27.40 ± 2.17	33.24 ± 2.45	24.86 ± 2.08	5.86 ± 0.35	5.58 ± 0.33	5.10 ± 0.33	6.15 ± 0.27	5.16 ± 0.59	5.21 ± 0.67	6.55 ± 0.67	4.13 ± 0.48
14	24.42 ± 1.82	23.96 ± 2.18	29.61 ± 2.52	22.71 ± 2.15	6.28 ± 0.36	6.02 ± 0.34	5.64 ± 0.33	6.50 ± 0.27	4.26 ± 0.59	4.33 ± 0.67	5.40 ± 0.67	3.53 ± 0.49
15	20.93 ± 1.82	20.33 ± 2.18	24.70 ± 2.50	20.22 ± 2.13	6.73 ± 0.36	6.58 ± 0.34	6.34 ± 0.33	6.89 ± 0.27	3.44 ± 0.59	3.48 ± 0.67	4.23 ± 0.67	2.93 ± 0.49
16	17.64 ± 1.81	16.71 ± 2.17	18.83 ± 2.46	17.11 ± 2.08	7.18 ± 0.35	7.18 ± 0.33	7.04 ± 0.33	7.26 ± 0.27	2.72 ± 0.59	2.70 ± 0.67	3.12 ± 0.67	2.39 ± 0.48
17	14.74 ± 1.82	13.32 ± 2.19	13.03 ± 2.53	13.57 ± 2.17	7.57 ± 0.36	7.70 ± 0.34	7.56 ± 0.34	7.56 ± 0.27	2.13 ± 0.59	2.03 ± 0.68	2.19 ± 0.67	1.94 ± 0.49
18	12.37 ± 1.81	10.39 ± 2.18	8.60 ± 2.47	10.43 ± 2.10	7.87 ± 0.36	8.04 ± 0.33	7.76 ± 0.33	7.74 ± 0.27	1.69 ± 0.59	1.49 ± 0.68	1.52 ± 0.67	1.63 ± 0.49
19	10.58 ± 1.82	8.05 ± 2.19	6.39 ± 2.47	8.79 ± 2.10	8.02 ± 0.36	8.18 ± 0.33	7.65 ± 0.33	7.75 ± 0.27	1.39 ± 0.59	1.09 ± 0.68	1.14 ± 0.67	1.49 ± 0.49
20	9.35 ± 1.85	6.33 ± 2.22	6.37 ± 2.55	9.31 ± 2.18	8.00 ± 0.36	8.18 ± 0.34	7.33 ± 0.34	7.62 ± 0.27	1.24 ± 0.60	0.82 ± 0.69	1.02 ± 0.68	1.51 ± 0.49
21	8.58 ± 1.84	5.18 ± 2.21	7.65 ± 2.44	11.54 ± 2.06	7.78 ± 0.36	8.04 ± 0.33	6.91 ± 0.33	7.36 ± 0.27	1.19 ± 0.60	0.66 ± 0.69	1.09 ± 0.68	1.65 ± 0.49
22	8.14 ± 1.86	4.47 ± 2.23	9.05 ± 2.55	14.02 ± 2.16	7.37 ± 0.37	7.76 ± 0.35	6.45 ± 0.34	7.01 ± 0.28	1.23 ± 0.60	0.59 ± 0.69	1.27 ± 0.69	1.87 ± 0.50
23	7.88 ± 1.89	4.06 ± 2.29	9.67 ± 2.63	15.13 ± 2.25	6.78 ± 0.37	7.28 ± 0.35	5.93 ± 0.35	6.58 ± 0.28	1.32 ± 0.62	0.58 ± 0.72	1.49 ± 0.70	2.09 ± 0.51
24	7.72 ± 2.12	3.83 ± 2.62	9.30 ± 2.51	14.26 ± 2.09	6.08 ± 0.40	6.60 ± 0.35	5.33 ± 0.34	6.10 ± 0.30	1.44 ± 0.71	0.60 ± 0.85	1.71 ± 0.78	2.29 ± 0.57
25	7.59 ± 3.02	3.66 ± 3.67	8.32 ± 3.89	12.04 ± 3.28	5.31 ± 0.58	5.80 ± 0.53	4.68 ± 0.52	5.59 ± 0.44	1.58 ± 0.99	0.64 ± 1.15	1.93 ± 1.11	2.47 ± 0.81
